# Upregulation of miR-665 promotes apoptosis and colitis in inflammatory bowel disease by repressing the endoplasmic reticulum stress components XBP1 and ORMDL3

**DOI:** 10.1038/cddis.2017.76

**Published:** 2017-03-23

**Authors:** Manying Li, Shenghong Zhang, Yun Qiu, Yao He, Baili Chen, Ren Mao, Yi Cui, Zhirong Zeng, Minhu Chen

**Affiliations:** 1 Division of Gastroenterology, The First Affiliated Hospital, Sun Yat-sen University, Guangzhou 510080, People's Republic of China

## Abstract

MicroRNAs are critical post-transcriptional regulators of gene expression and key mediators of pathophysiology of inflammatory bowel disease (IBD). This study is aimed to study the role of miR-665 in the progression of IBD. Real-time PCR analysis was used to determine miR-665 expression in 89 freshly isolated IBD samples and dextran sulfate sodium (DSS)-induced colonic mucosal tissues. The role of miR-665 in inducing apoptosis and colitis were examined by Annexin V, TUNEL (terminal deoxynucleotidyl transferase dUTP nick-end labeling) staining, colony formation *in vitro* and DSS-induced colitis mice model *in vivo.* Moreover, luciferase reporter assay, western blot analysis and microribonucleoprotein immunoprecipitation were performed to determine that miR-665 directly repressed XBP1 (X-box-binding protein-1) and ORMDL3 expression. Herein, our results revealed that miR-665 was markedly upregulated in active colitis. Gain-of-function and loss-of-function studies showed that ectopic expression of miR-665 promoted apoptosis under different inflammatory stimuli. Importantly, delivery of miR-665 mimic promoted, while injection of antagomiR-665 markedly impaired DSS-induced colitis *in vivo*. Mechanistically, we demonstrated that miR-665 induced apoptosis by inhibiting XBP1 and ORMDL3. Taken together, our findings reveal a new regulatory mechanism for ER stress signaling and suggest that miR-665 might be a potential target in IBD therapy.

Inflammatory bowel disease (IBD) is a complex disease characterized by chronic or recurrent inflammation in the gastrointestinal tract. The chief types of IBD are ulcerative colitis (UC) and Crohn's disease (CD). Although IBD is rarely fatal on its own, it is often not medically curable owing to the chronic inflammation, and might increase risk of colorectal cancer. Recent progress in understanding IBD pathobiology has shown that genetically based interactions between environmental and/or epithelial barrier dysfunctions cause persistent activation of the innate and adaptive immune responses.^1^ Notably, recent studies have provided valid evidence linking the endoplasmic reticulum (ER) stress to the pathogenesis of IBD.^2^

ER stress is a cellular process triggered by a variety of conditions that disturb protein folding in the ER. To clear unfolded proteins, the unfolded protein response (UPR) restores ER homeostasis. The X-box-binding protein-1 (XBP1), a potent inducer of a subset of UPR target genes, is required for the constitutive maintenance of ER function in all cell types, and XBP1 deletion in intestinal epithelial cells (IECs) results in spontaneous enteritis.^3, 4^ Furthermore, the ORMDL3 protein is located primarily in the ER and regulates Ca^2+^ uptake from the cytosol and ER-mediated Ca^2+^ signaling.^5, 6^ Recently, genome-wide association studies identified ORMDL3 as another genetic risk factor of CD and UC.^7^ Loss of XBP1 or ORMDL3 expression has been reported to promote colitis and increase hypersensitivity to cytokine-induced apoptosis via c-Jun N-terminal kinase (JNK) signaling.^2, 3, 8^ Regarding their critical roles in ER stress and pathogenesis of IBD, better understanding of the regulation of XBP1 and ORMDL3 might provide new clues for the prognosis and therapy of IBD.

Owning to its role of simultaneously repressing a variety of target genes by interaction with their 3′-UTR elements of mRNA, microRNA (miRNA) has been involved in the development and progression of IBD.^9, 10, 11^ Of note, recent studies suggest that miRNAs regulate ER stress and induce apoptosis.^12, 13^ For example, miR-15b-5p repressed UPR signaling by suppressing Rab1A and accelerated apoptosis in human hepatocellular carcinoma.^14^ However, whether miRNA-mediated ER stress alteration contributes to IBD progression remains unknown.

Notably, a recent study comparing the miRNA expression alteration identified that miR-665 was significantly upregulated in mucosal colonic biopsies of active UC;^15^ however, its role remains unclear. Herein, using the TargetScan algorithm, we found that both *XBP1* and *ORMDL3* are theoretically target genes of miR-665. Furthermore, we demonstrated that miR-665 downregulated XBP1 and ORMDL3 expression by directly targeting their 3′-UTRs, consequently resulting in the activation of JNK. Upregulation of miR-665 promotes apoptosis and dextran sulfate sodium (DSS)-induced colitis both *in vivo* and *in vitro*, while inhibition of miR-665 induces opposite effects. Taken together, these results suggest that miR-665 have an important role in the regulation of ER stress and might represent a therapeutic target for IBD.

## Results

### miR-665 is markedly upregulated in the inflamed mucosa of patients with IBD and DSS-induced colitis in mice

**Figure 1:**
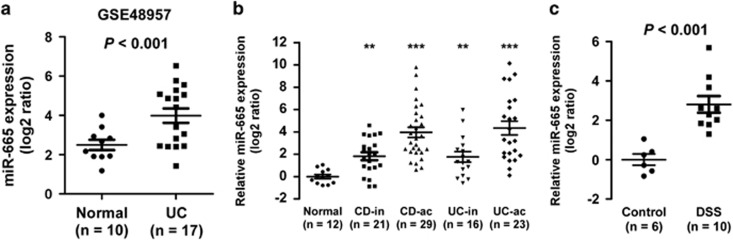
miR-665 is markedly upregulated in the inflamed mucosa of patients with IBD and DSS-induced colitis in mice. (**a**) The miR-665 levels remained low in normal colonic mucosal biopsies but became markedly higher in patients with UC assessed by a published microarray (NCBI/GEO/GSE48957; *n*=27, including 10 normal controls, 17 UC samples). *P*<0.001. (**b**) Real-time PCR analysis of miR-665 expression in 50 CD (inactive CD, *n*=21; active CD, *n*=29) and 39 UC (inactive UC, *n*=16; active UC, *n*=23) samples compared with normal controls. Transcript levels were normalized to *U6* expression. Lines denote the means±S.D. **P<0.05, ***P<0.001, two-tailed Student's *t*-test. (**c**) Real-time PCR analysis of miR-665 expression in DSS-induced colitis mice and controls. Transcript levels were normalized to *U6* expression. Lines denote the means±S.D.Two-tailed Student's *t*-test

MiRNAs are emerging as important regulators in IBD pathogenesis. By analyzing a publically published miRNA expression profile (NCBI/GEO/GSE48957; *n*=27, including 10 normal controls and 17 UC samples),^15^ we found that miR-665 levels remained low in normal colonic mucosal biopsies but became about fourfold elevated in patients with UC (Figure 1a). We then validated miR-665 expression in IBD tissues using real-time PCR. As expected, miR-665 expression was substantially increased, with ~4–16-fold upregulation in CD (inactive CD, *n*=21; active CD, *n*=29) and UC (inactive UC, *n*=16; active UC, *n*=23) subgroups compared with normal controls, and further elevated in the patients with active colitis (Figure 1b). Moreover, miR-665 expression was also increased by ~6-fold in DSS-induced colonic mucosal tissues. Thus, these results suggest that upregulation of miR-665 may contribute to IBD progression.

### miR-665 sensitizes inflammation-induced apoptosis *in vitro*

**Figure 2:**
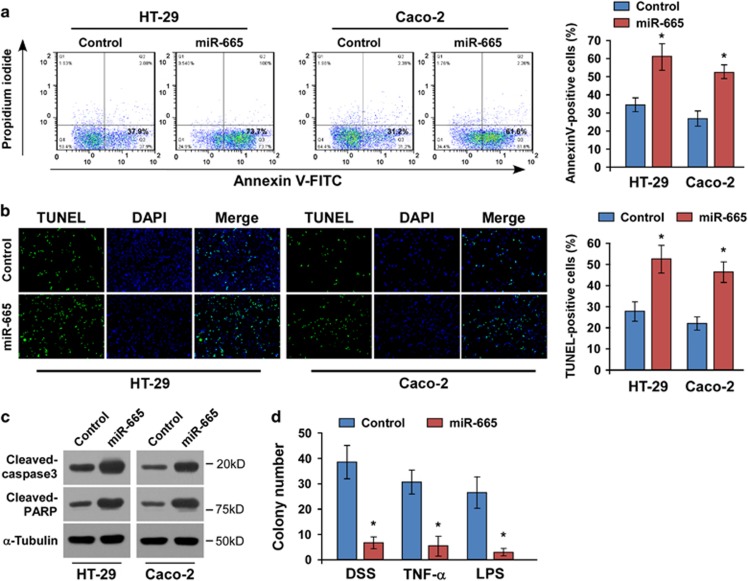
miR-665 sensitizes inflammation-induced apoptosis *in vitro.* (**a**) Annexin V-fluorescein isothiocyanate (FITC)/propidium iodide (PI) staining of the indicated cells treated with 2% DSS for 24 h. (**b**) Representative micrographs (left) and quantifications (right) of TUNEL-positive cells following 24 h of 2% DSS treatment. (**c**) Western blotting of cleaved caspase-3 and PARP expression. *α*-Tubulin was used as the loading control. (**d**) Quantification of colonies formed in the miR-665-transfected cells and controls in the presence of 2% DSS, TNF-*α* (20 ng/ml) and LPS (1 *μ*g/ml). Error bars represent the means±S.D. of three independent experiments. *P<0.05

Since IBD is characterized by diffuse mucosal inflammation and cell apoptosis in both CD and UC, we speculated that miR-665 might have a role in apoptosis. We first exogenously overexpressed miR-665 via miR-665 mimic transfection (Supplementary Figure 1). Indeed, Annexin V and TUNEL (terminal deoxynucleotidyl transferase dUTP nick-end labeling) staining assays demonstrated that ectopic expression of miR-665 increased the apoptotic rates of HT-29 and Caco-2 cells by sensitization to the chemoagent DSS (Figures 2a and b). Moreover, the effect of miR-665 on promoting apoptosis was confirmed by examining the cleavage profiles of procaspase-3 and poly (ADP-ribose) polymerase (PARP) in HT-29 and Caco-2 cells. As shown in Figure 2c, the cleavages of both caspase-3 and PARP were increased in the miR-665-overexpressing cells treated with DSS. Importantly, the colony formation assay indicated that miR-665 overexpression induced apoptosis of HT-29 cells in the presence of DSS, tumor necrosis factor-*α* (TNF-*α*) and lipopolysaccharide (LPS) compared with the controls (Figure 2d).

**Figure 3:**
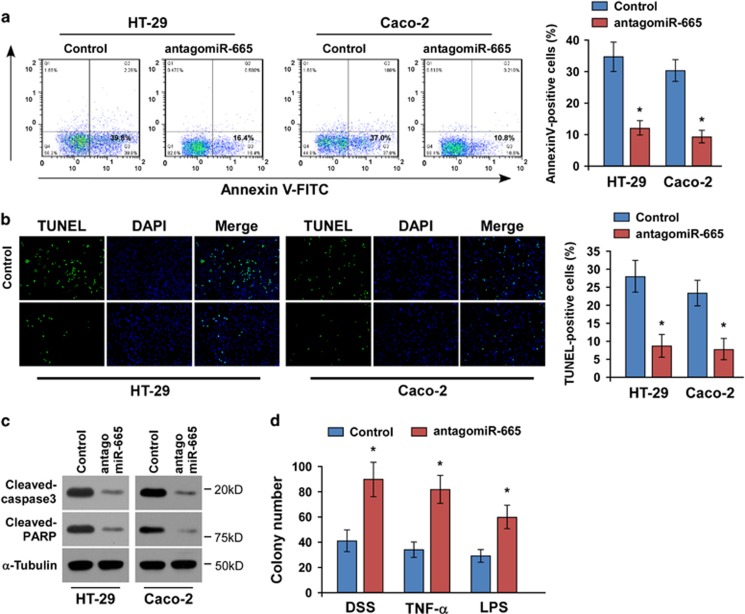
Silencing of miR-665 protects against cell death *in vit*ro. (**a**) Cells were treated with DSS for 24 h and stained with Annexin V-fluorescein isothiocyanate (FITC)/PI. (**b**) Silencing of miR-665 reduced the number of TUNEL-positive cells following 24 h of treatment with DSS. (**c**) Western blotting of cleaved caspase-3 and PARP expression. *α*-Tubulin was used as the loading control. (**d**) Quantification of cell colonies formed in the antagomiR-665-transfected cells and controls in the presence of DSS, TNF-*α* (20 ng/ml) and LPS (1 *μ*g/ml). Error bars represent the means±S.D. of three independent experiments. *P<0.05

Conversely, the role of miR-665 in IBD was further examined by endogenously silencing miR-665 through antagomiR-665 transfection (Supplementary Figure 1). We found that silencing miR-665 reduced the apoptotic rates of HT-29 and Caco-2 cells under DSS treatment (Figure 3a and b). Consistently, the cleavages of both caspase-3 and PARP were suppressed in the miR-665-silenced cells treated with DSS (Figure 3c). Moreover, the number of colonies formed by miR-665-silenced cells was significantly greater compared with controls upon treatment with DSS, TNF-*α* and LPS (Figure 3d). Taken together, our results indicate that upregulation of miR-665 promotes inflammation-induced apoptosis *in vitro*.

### Antagonizing miR-665 impairs DSS-induced colitis *in vivo*

**Figure 4:**
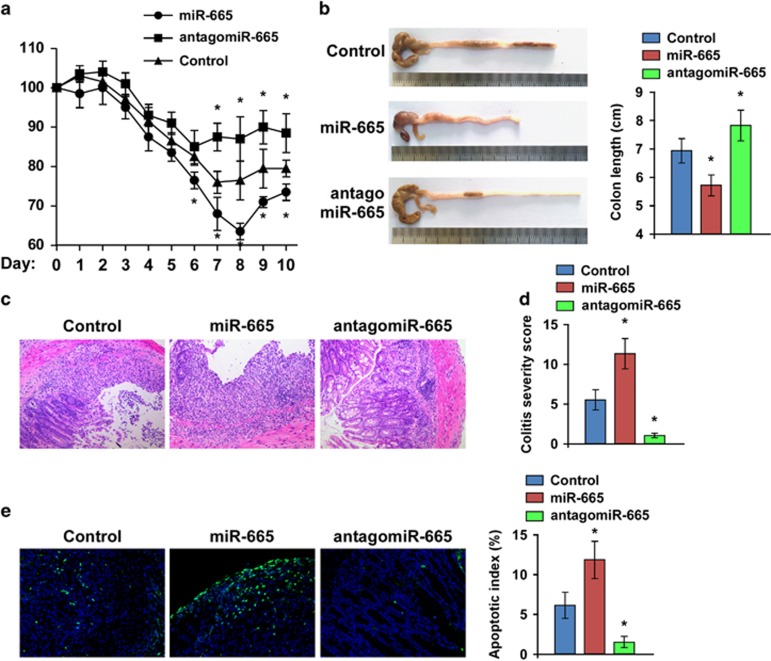
Antagonizing miR-665 inhibits DSS-induced colitis *in vivo.* (**a**) Body weight changes are shown as percentages of the baseline value and are the means±S.E.M., *n*=8. (**b**) Ten days after DSS treatment, colon tissues were examined. (**c**) Mucosal histology was examined by H&E staining. (**d**) Colitis severity score in each group. (**e**) The apoptotic index (right) was determined using the percentage of TUNEL-positive cells (left). Error bars represent the means±S.D. of three independent experiments. *P<0.05

We next examined the promotive effect of miR-665 on IBD *in vivo*, and assessed whether antagonizing miR-665 could impair DSS-induced colitis. Mice were intraperitoneally injected with miR-665 mimic, antagomiR-665 or control three times a week for 2 weeks. One week after injection, the mice were subjected to DSS-induced colitis for 10 days. We observed the greatest body weight loss in mice injected with the miR-665 mimic, whereas antagomiR-665 injection significantly inhibited DSS-induced body weight loss (Figure 4a). Furthermore, mice injected with miR-665 had shorter colon lengths, loss of crypt structure, ulceration, infiltration of inflammatory cells and a higher apoptosis index compared with controls (Figures 4b–e). Importantly, inhibition of miR-665 robustly impaired DSS-induced colon shortening, colitis and cell death *in vivo* (Figures 4b–e). These results suggest that miR-665 promotes DSS-induced colitis, and that antagonizing miR-665 rescues cells from these effects.

### miR-665 targets ER stress components XBP1 and ORMDL3

**Figure 5:**
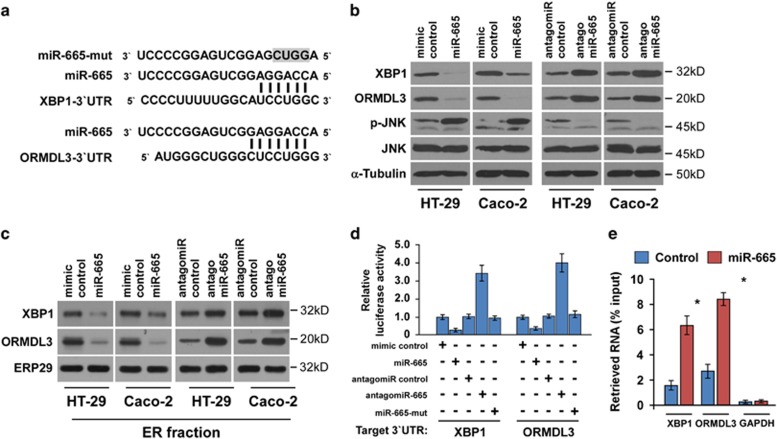
miR-665 targets ER stress components XBP1 and ORMDL3. (**a**) The predicted miR-665 target sequence in the 3′-UTRs of *XBP1* and *ORMDL3*. The mutated miR-665 (miR-665-mutant) containing four altered nucleotides in the miR-665 seed sequence is indicated. (**b**) Western blots of XBP1, ORMDL3, p-JNK and JNK expression. *α*-Tubulin served as the loading control. (**c**) Western blots of XBP1 and ORMDL3 expression in the ER fractions. ERP29 served as the marker of ER. (**d**) Luciferase assay of cells transfected with pGL3-XBP1-3′-UTR or pGL3-ORMDL3-3′-UTR reporter with miR-665 mimic, antagomiR-665, mimic control, antagomiR control or miR-665-mutant. (**e**) MiRNP IP assay showing the association between miR-665 and *XBP1* and *ORMDL3* transcripts in HT-29 cells. *GAPDH* served as the negative control. Error bars represent the means±S.D. of three independent experiments. *P<0.05

Recent advances have revealed that the ER stress pathway is a primitive cellular pathway that maintains cellular viability and homeostasis of the colon. Interestingly, using the publicly available algorithm TargetScan, we found that the core components of the UPR signaling pathway, *XBP1* and *ORMDL3*, may be potential targets of miR-665 (Figure 5a). Western blotting analysis revealed that miR-665 overexpression significantly suppressed XPB1 and ORMDL3 expression levels, but miR-665 inhibition increased their expression (Figure 5b). Meanwhile, we found that the accumulation of XBP1 and ORMDL3 in cellular ER was markedly reduced by overexpression of miR-665, but increased by its inhibition (Figure 5c). However, real-time PCR analysis revealed that the mRNA levels of XBP1 and ORMDL3 were not significantly changed upon alteration of miR-665 (Supplementary Figure 2), suggesting that miR-665 inhibits XBP1 and ORMDL3 expression by inducing translation repression.

Previous study has found that downregualtion of XBP1 promotes apoptosis via JNK activation.^3^ Consistently, we found that ectopic expression of miR-665 increased the phosphorylation of JNK (p-JNK), while inhibition of miR-665 decreased its phosphorylation, suggesting that miR-665 promotes cell apoptosis by activating JNK (Figure 5b). Furthermore, luciferase assay showed that miR-665 overexpression attenuated the reporter activities driven by the 3′-UTRs of XPB1 and ORMDL3, whereas inhibition of miR-665 elevated these activities (Figure 5d). However, ectopic expression of the miR-665 mutant did not have repressive effects on the reporter activities (Figure 5d). Moreover, a microribonucleoprotein (miRNP) immunoprecipitation (IP) assay revealed a selective association of miR-665 with XBP1 and ORMDL3 but not with GAPDH (Figure 5e), further indicating the specific effects of miR-665 on these targets. These results suggest that miR-665 directly targeted XBP1 and ORMDL3.

### XBP1 and ORMDL3 are functional effectors for miR-665-induced apoptosis

**Figure 6:**
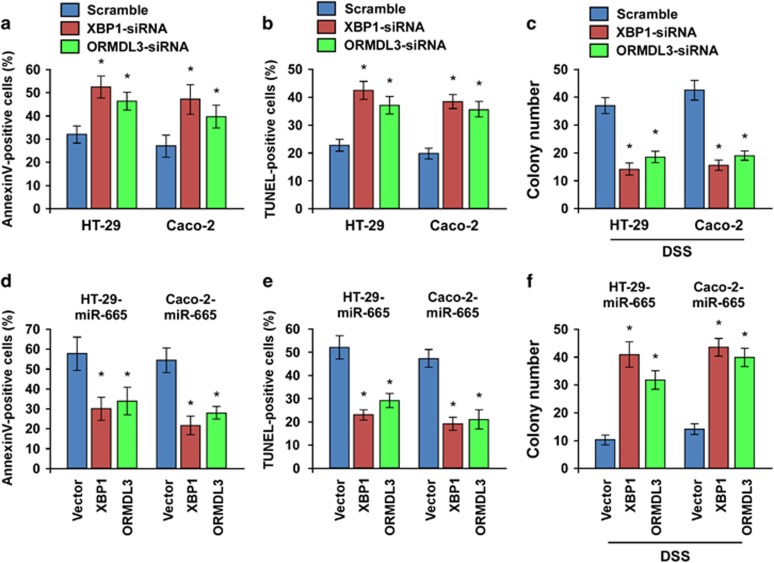
XBP1 and ORMDL3 are functional effectors for miR-665-induced apoptosis. (**a** and **b**) Annexin V (**a**) and TUNEL (**b**) staining assays indicating that silencing of XBP1 or ORMDL3 promoted cellular apoptosis of colon cells. (**c**) Quantification of colonies formed in the XBP1- or ORMDL3-silenced cells in the presence of DSS. (**d** and **e**) Annexin V (**d**) and TUNEL (**e**) staining assays indicating that both XBP1 and ORMDL3 expression abrogated miR-665-induced apoptosis. (**f**) Quantification of colonies in indicated cells. Error bars represent the means±S.D. of three independent experiments. *P<0.05

We then explored the functional significance of XBP1 and ORMDL3 repression in the apoptosis of colon cells. As shown in Figures 6a and b, either silencing of XBP1 or ORMDL3 in HT-29 and Caco-2 colon cells increased the sensitivity of DSS-induced apoptosis. Meanwhile, XBP1- or ORMDL3-silenced cells formed more colonies under DSS treatment (Figure 6c). Furthermore, we found that restoration of both XBP1 and ORMDL3 expression abrogated the stimulatory effect of miR-665 on cellular apoptosis, as indicated by Annexin V, TUNEL and colony formation assays (Figures 6d–f). On the other hand, either silencing of XBP1 or ORMDL3 promoted cellular apoptosis in miR-665-inhibited cells (Supplementary Figure 3A–C).

Collectively, these findings indicate that upregulation of miR-665 promotes IBD progression by inactivating ER stress signaling via targeting XBP1 and ORMDL3, and miR-665 might be a potential therapeutic target.

## Discussion

ER stress is observed in many human diseases, such as inflammation, neurodegenerative diseases, metabolic diseases and cancers, and it is identified that the UPR, a three-pronged signaling axis, to preserve ER homeostasis.^16^ These stress responses are involved in multiple cellular processes, including protein synthesis, degradation rates, folding and secretion, and then induce to cell migration, cell transformation, angiogenesis and cell death.^17^ Notably, for the intestinal epithelium, ER stress is characteristically induced by primary (genetic) causes associated with the UPR, such as XBP1, ORMDL3 and ARG2, or secondary (environmental) factors, including bacterial toxins (e.g., subAB), mucins (e.g., MUC2), autophagic proteins (e.g., ATG16L1) and cytokines (e.g., TNF-*α* and interleukin-10), and the interplay of these factors initiates the cascades of inflammation that can cause or exacerbate homeostatic ER stress.^18^ Moreover, cell death is always triggered if the homeostasis cannot be re-established. Thus, ER stress has an important role in the development of intestinal inflammation associated with IBD. Although great advances have been made in the understanding of ER stress, the regulatory mechanism for deregulation of ER stress in IBD remains unclear.^19^ In this study, we found that miR-665 was upregulated in IBD samples, and substantially repressed the components of ER stress, XBP1 and ORMDL3, leading to enhancing apoptosis by activation of JNK. Therefore, our study presents a novel mechanism for ER stress signaling modulation, which may be a promising therapeutic target for IBD.

Recent advances have provided new insights into understanding of ER stress-induced cell death mechanisms, and it is suggested that some miRNAs act as the suppressors of ER stress-mediated cell death.^20^ Although the interplay of miRNAs and ER stress response needs further investigation, we identified that miR-665 targeted to XBP1 and ORMDL3, which were in different signaling pathways of ER stress responses, then activated the expression of JNK, consequently resulting in apoptosis. Indeed, it is reported that 4-phenyl butyric acid (an FDA-approved drug for urea-cycle disorders) and taurine-conjugated ursodeoxycholic acid (TUDCA) reduce ER stress *in vivo* using murine models of obesity and type 2 diabetes.^21^ We found that several clinical trials have focused on the role of TUDCA in the subjects who are type 1 diabetes or protease-inhibitor associated insulin resistance. Interestingly, oral administration of TUDCA ameliorated inflammation in a rodent model of small intestinal inflammation, then ER stress may be an important mechanism in the pathology of IBD.^22^ In our study, miR-665 expression was markedly upregulated in experimental colitis, but antagonizing miR-665 reduces the severity of colitis. Therefore, our results suggest that miR-665 may represent as a potential therapeutic target for IBD.

As we know, p53 is the main gatekeeper of cellular apoptosis. However, the ER stress is found to induce apoptosis mainly through activation of the JNK signaling, which in turn regulates the expression of BCL2 and BAX, leading to mitochondrion-mediated cell death.^23^ In this context, we here used the p53-mutant HT-29 and p53-null Caco-2 cells to investigate the alteration of ER stress signaling by miR-665. Notably, we found that miR-665 reduced XBP1 and ORMDL3 expression, and increased the activity of JNK, leading to the increased sensitivity of cellular apoptosis by inflammatory factors. Thus, these findings revealed that the miR-665/ER/JNK axis had an important role in IBD progression independent of p53.

Recent evidence indicated that miR-665 was upregulated in intestinal gastric adenocarcinoma.^24^ It is reported that IBD has the risk of developing colitis associated cancer (CAC), and the increasing cancer incidence has been up to 20%.^25^ Polytarchou *et al*.^26^ recently have found that miR-214 was overexpressed by IL-6/STAT3 signaling activation, and modulated the expressions of phosphatase and tensin homolog (PTEN), PDZ and LIM domain 2 (PDLIM2) and phosphorylation of AKT, which induced the activation of nuclear factor-*κ*B (NF-*κ*B). Moreover, they demonstrated that the signaling correlates with the progression to colorectal cancer.^26^ Meanwhile, the NF-*κ*B transcription factor family is an important initiator of inflammation by ER stress, leading to the production of proinflammatory TNF-*α* and exacerbating stress-induced cell death.^27^ Interestingly, our results indicated that ectopic expression of miR-665 promoted apoptosis under different inflammatory stimuli, such as TNF-*α*. However, whether miR-665 might have a role in the development of CAC through the effects of inflammatory factors remains unknown. Efforts need to be made to examine the crucial role of miR-665 in the progression of CAC.

Microbiota is regarded as a main cause for IBD.^28^ Interestingly, miR-665 was reported to be induced by microbiota, which in turn facilitated the infection by downregulating Abcc3 in host cells,^29^ suggesting that miR-665 might be an important mediator in microbiota-induced IBD. On the other hand, ER stress has various mediators in immunity including proinflammatory cytokine production. Notably, by analyzing the miR-665 promoter region using the CONSITE program, we found several typical NF-*κ*B binding sites, suggesting that miR-665 might be regulated by the inflammatory NF-*κ*B signaling pathway. Thus, miR-665 might induce a positive feedback regulation in the miR-665/ER/NF-*κ*B loop, leading to the chronic inflammation in the gastrointestinal tract. While these hypotheses are great directions to pursue, however, it is beyond the scope of our current project and therefore will be investigated in our future research.

In conclusion, our study demonstrated that miR-665 is a critical regulator of ER stress signaling, and promotes the pathogenesis in IBD. Understanding the precise role of miR-665 in IBD pathogenesis and in the ER stress response promises to increase our knowledge of the biological basis of inflammation development and may also facilitate the development of new therapeutic strategies against IBD.

## Materials and Methods

### Patient biopsies and ethical considerations

Patients with IBD and non-IBD control subjects were recruited with written informed consent for this study at the First Affiliated Hospital of Sun Yat-sen University. And, the Hospital Ethics Committee approved the study protocol. The inflamed biopsy specimens were obtained from patients diagnosed with CD or UC following the standard clinical, endoscopic and histological criteria, including 29 patients with active CD and 21 patients in remission and 23 patients with active UC and 16 patients in remission. The severity of diseases was assessed according to the international standard criteria, such as the Crohn's disease activity index for the diagnosis of patients with CD, and Mayo scores for patients with UC. Control samples were obtained from patients undergoing colonoscopy as part of a routine program of polyp surveillance without active gastrointestinal pathology.

### Western blotting analysis

Cells were harvested in cell lysis buffer (Cell Signaling Technology, Danvers, MA, USA) and heated for 5 min at 100 °C. Equal quantities of denatured protein samples were resolved on 10% SDS-polyacrylamide gels and were then transferred onto polyvinylidene difluoride membranes (Roche, Basel, Switzerland). After blocking with 5% non-fat dry milk in TBS/0.05% Tween-20, the membranes were incubated with a specific primary antibody followed by a horseradish peroxidase-conjugated secondary antibody. Proteins were visualized using ECL reagents (Pierce, Rockford, IL, USA). Antibodies against cleaved caspase-3, cleaved PARP, XBP1, ORMDL3, ERP29, p-JNK and JNK were purchased from Abcam (Cambridge, MA, USA). The membranes were stripped and reprobed with an anti-*α*-tubulin antibody (Sigma-Aldrich, St. Louis, MO, USA) as the loading control.

### miRNA extraction and real-time quantitative PCR

Total miRNA from fresh biopsy colon tissues was extracted using a mirVana miRNA Isolation Kit (Ambion, Austin, TX, USA) according to the manufacturer's instructions. We synthesized cDNA from 10 ng total RNA using a TaqMan miRNA Reverse Transcription Kit (Applied Biosystems, Foster City, CA, USA), and we quantified the expression levels of miR-665 using an miRNA-specific TaqMan MiRNA Assay Kit (Applied Biosystems). Expression of miRNA was defined based on the Ct, and relative expression levels were calculated as 2^−[(Ct of miR-665)–(Ct of U6)]^ after normalization with reference to the expression of U6 small nuclear RNA.

### TUNEL assay

Apoptotic DNA fragmentation was examined using an *in situ* DeadEnd Fluorometric TUNEL System Assay Kit (Promega, Madison, WI, USA) according to the manufacturer's protocol. Briefly, cells were plated in 24-well flat-bottom plates and treated with 2% DSS for 24 h. Cells were fixed in 4% paraformaldehyde at 4 °C for 30 min, permeabilized in 0.1% Triton X-100 and labeled with fluorescein-12-dUTP using terminal deoxynucleotidyl transferase. The localized green fluorescence of the apoptotic cells from the fluorescein-12-dUTP was detected by fluorescence microscopy (Axiovert 100 M; Zeiss, Oberkochen, Germany).

### The DSS-induced colitis model

C57BL/6 mice (6–8 weeks old, 18–20 g) were purchased from the Experimental Animal Center of the Guangzhou University of Chinese Medicine and housed in barrier facilities on a 12-h light–dark cycle. The Institutional Animal Care and Use Committee of Sun Yat-sen University approved all experimental procedures. The mice were randomly assigned to groups (*n*=8 per group). The mice in the groups were injected intraperitoneally with 100 *μ*l miR-665 mimic, antagomiR-665 or negative control (diluted in PBS at 2 mg/ml) three times per week for 2 weeks. After 1 week, the mice were subjected to treatment with 2% DSS for 10 days. The mice weights were calculated. Animals were then killed, and the colon tissues were excised and paraffin-embedded. Sections of colon tissues were subjected to hematoxylin and eosin (H&E) staining and the TUNEL assay. The extent of inflammation was measured and scored as described previously,^30^ and the apoptotic index was measured based on the percentage of TUNEL-positive cells.

### Luciferase assay

Cells (4 × 10^4^) were seeded in triplicate in 24-well plates and cultured for 24 h. Cells were transfected with 100 ng pGL3-XBP1-3′-UTR or pGL3-ORMDL3-3′-UTR luciferase plasmid plus 5 ng pRL-TK *Renilla* plasmid (Promega) using Lipofectamine 2000 (Invitrogen, Carlsbad, CA, USA) according to the manufacturer's recommendations. Luciferase and *Renilla* signals were measured 36 h after transfection using a Dual Luciferase Reporter Assay Kit (Promega) according to the manufacturer's protocol.

### miRNP immunoprecipitation

Cells were co-transfected with HA-Ago1 together with 100 nM miR-665, followed by HA-Ago1 immunoprecipitation using an antibody against HA. Real-time PCR analysis of the immunoprecipitated material was used to test the association of the mRNA of XBP1 and ORMDL3 with the RISC complex.

### Statistical analysis

All statistical analyses were performed using the SPSS version 19.0 (SPSS lnc., Chicago, IL, USA) statistical software package. Data are expressed as the means±S.D. Comparisons between groups were performed with an unpaired two-tailed Student's *t*-test. In all cases, a *P*-value<0.05 was considered statistically significant.

## References

[bib1] Pedersen J, LaCasse EC, Seidelin JB, Coskun M, Nielsen OH. Inhibitors of apoptosis (IAPs) regulate intestinal immunity and inflammatory bowel disease (IBD) inflammation. Trends Mol Med 2014; 20: 652–665. 25282548 10.1016/j.molmed.2014.09.006

[bib2] Kaser A, Tomczak M, Blumberg RS. 'ER stress(ed out)!': Paneth cells and ischemia–reperfusion injury of the small intestine. Gastroenterology 2011; 140: 393–396. 21172333 10.1053/j.gastro.2010.12.015 PMC4594951

[bib3] Kaser A, Lee AH, Franke A, Glickman JN, Zeissig S, Tilg H et al. XBP1 links ER stress to intestinal inflammation and confers genetic risk for human inflammatory bowel disease. Cell 2008; 134: 743–756. 18775308 10.1016/j.cell.2008.07.021 PMC2586148

[bib4] Cao SS, Wang M, Harrington JC, Chuang BM, Eckmann L, Kaufman RJ. Phosphorylation of eIF2alpha is dispensable for differentiation but required at a posttranscriptional level for paneth cell function and intestinal homeostasis in mice. Inflamm Bowel Dis 2014; 20: 712–722. 24577114 10.1097/MIB.0000000000000010

[bib5] Kaser A, Martinez-Naves E, Blumberg RS. Endoplasmic reticulum stress: implications for inflammatory bowel disease pathogenesis. Curr Opin Gastroenterol 2010; 26: 318–326. 20495455 10.1097/MOG.0b013e32833a9ff1 PMC4592137

[bib6] Adolph TE, Niederreiter L, Blumberg RS, Kaser A. Endoplasmic reticulum stress and inflammation. Dig Dis 2012; 30: 341–346. 22796794 10.1159/000338121 PMC3423328

[bib7] Hoefkens E, Nys K, John JM, Van Steen K, Arijs I, Van der Goten J et al. Genetic association and functional role of Crohn disease risk alleles involved in microbial sensing, autophagy, and endoplasmic reticulum (ER) stress. Autophagy 2013; 9: 2046–2055. 24247223 10.4161/auto.26337

[bib8] Hsu KJ, Turvey SE. Functional analysis of the impact of ORMDL3 expression on inflammation and activation of the unfolded protein response in human airway epithelial cells. Allergy Asthma Clin Immunol 2013; 9: 4. 23369242 10.1186/1710-1492-9-4 PMC3651386

[bib9] Ambros V. The functions of animal microRNAs. Nature 2004; 431: 350–355. 15372042 10.1038/nature02871

[bib10] Wang H, Chao K, Ng SC, Bai AH, Yu Q, Yu J et al. Pro-inflammatory miR-223 mediates the cross-talk between the IL23 pathway and the intestinal barrier in inflammatory bowel disease. Genome Biol 2016; 17: 58. 27029486 10.1186/s13059-016-0901-8 PMC4815271

[bib11] Yu Q, Zhang S, Chao K, Feng R, Wang H, Li M et al. E3 ubiquitin ligase RNF183 is a novel regulator in inflammatory bowel disease. J Crohn's Colitis 2016; 10: 713–725. 26818663 10.1093/ecco-jcc/jjw023

[bib12] Chhabra R, Dubey R, Saini N. Gene expression profiling indicate role of ER stress in miR-23a~27a~24-2 cluster induced apoptosis in HEK293T cells. RNA Biol 2011; 8: 648–664. 21593605 10.4161/rna.8.4.15583

[bib13] Yang F, Zhang L, Wang F, Huo XS, Yin YX, Wang YQ et al. Modulation of the unfolded protein response is the core of microRNA-122-involved sensitivity to chemotherapy in hepatocellular carcinoma. Neoplasia (New York, NY) 2011; 13: 590–600. 10.1593/neo.11422 PMC3132845 21750653

[bib14] Yang Y, Hou N, Wang X, Wang L, Chang S, He K et al. miR-15b-5p induces endoplasmic reticulum stress and apoptosis in human hepatocellular carcinoma, both *in vitro* and *in vivo*, by suppressing Rab1A. Oncotarget 2015; 6: 16227–16238. 26023735 10.18632/oncotarget.3970 PMC4599266

[bib15] Van der Goten J, Vanhove W, Lemaire K, Van Lommel L, Machiels K, Wollants WJ et al. Integrated miRNA and mRNA expression profiling in inflamed colon of patients with ulcerative colitis. PLoS ONE 2014; 9: e116117. 25546151 10.1371/journal.pone.0116117 PMC4278881

[bib16] Chevet E, Hetz C, Samali A. Endoplasmic reticulum stress-activated cell reprogramming in oncogenesis. Cancer Discov 2015; 5: 586–597. 25977222 10.1158/2159-8290.CD-14-1490

[bib17] Cao SS. Epithelial ER stress in Crohn's disease and ulcerative colitis. Inflamm Bowel Dis 2016; 22: 984–993. 26950312 10.1097/MIB.0000000000000660

[bib18] Kaser A, Blumberg RS. Autophagy, microbial sensing, endoplasmic reticulum stress, and epithelial function in inflammatory bowel disease. Gastroenterology 2011; 140: 1738–1747. 21530740 10.1053/j.gastro.2011.02.048 PMC4592160

[bib19] Ron D, Walter P. Signal integration in the endoplasmic reticulum unfolded protein response. Nat Rev Mol Cell Biol 2007; 8: 519–529. 17565364 10.1038/nrm2199

[bib20] Sano R, Reed JC. ER stress-induced cell death mechanisms. Biochim Biophys Acta 2013; 1833: 3460–3470. 23850759 10.1016/j.bbamcr.2013.06.028 PMC3834229

[bib21] Fritz T, Niederreiter L, Adolph T, Blumberg RS, Kaser A. Crohn's disease: NOD2, autophagy and ER stress converge. Gut 2011; 60: 1580–1588. 21252204 10.1136/gut.2009.206466 PMC3897479

[bib22] Cao SS. Endoplasmic reticulum stress and unfolded protein response in inflammatory bowel disease. Inflamm Bowel Dis 2015; 21: 636–644. 25581827 10.1097/MIB.0000000000000238

[bib23] Szegezdi E, Logue SE, Gorman AM, Samali A. Mediators of endoplasmic reticulum stress-induced apoptosis. EMBO Rep 2006; 7: 880–885. 16953201 10.1038/sj.embor.7400779 PMC1559676

[bib24] Chen J, Sun D, Chu H, Gong Z, Zhang C, Gong B et al. Screening of differential microRNA expression in gastric signet ring cell carcinoma and gastric adenocarcinoma and target gene prediction. Oncol Rep 2015; 33: 2963–2971. 25964059 10.3892/or.2015.3935

[bib25] Francescone R, Hou V, Grivennikov SI. Cytokines, IBD, and colitis-associated cancer. Inflamm Bowel Dis 2015; 21: 409–418. 25563695 10.1097/MIB.0000000000000236 PMC4481731

[bib26] Polytarchou C, Hommes DW, Palumbo T, Hatziapostolou M, Koutsioumpa M, Koukos G et al. MicroRNA214 is associated with progression of ulcerative colitis, and inhibition reduces development of colitis and colitis-associated cancer in mice. Gastroenterology 2015; 149: 981–992 e911. 26055138 10.1053/j.gastro.2015.05.057 PMC4584179

[bib27] Bettigole SE, Glimcher LH. Endoplasmic reticulum stress in immunity. Annu Rev Immunol 2015; 33: 107–138. 25493331 10.1146/annurev-immunol-032414-112116

[bib28] Kotanko P, Carter M, Levin NW. Intestinal bacterial microflora – a potential source of chronic inflammation in patients with chronic kidney disease. Nephrol Dial Transplant 2006; 21: 2057–2060. 16762961 10.1093/ndt/gfl281

[bib29] Dalmasso G, Nguyen HT, Yan Y, Laroui H, Charania MA, Ayyadurai S et al. Microbiota modulate host gene expression via microRNAs. PLoS ONE 2011; 6: e19293. 21559394 10.1371/journal.pone.0019293 PMC3084815

[bib30] Greten FR, Eckmann L, Greten TF, Park JM, Li ZW, Egan LJ et al. IKKbeta links inflammation and tumorigenesis in a mouse model of colitis-associated cancer. Cell 2004; 118: 285–296. 15294155 10.1016/j.cell.2004.07.013

