# Supplementary figures and images for: Upregulation of miR-665 promotes apoptosis and colitis in inflammatory bowel disease by repressing the endoplasmic reticulum stress components XBP1 and ORMDL3
Source: Cell Death Dis. 2017 Mar 23;8(3):e2699–. doi: 10.1038/cddis.2017.76 (PMC5386569; doi:10.1038/cddis.2017.76)

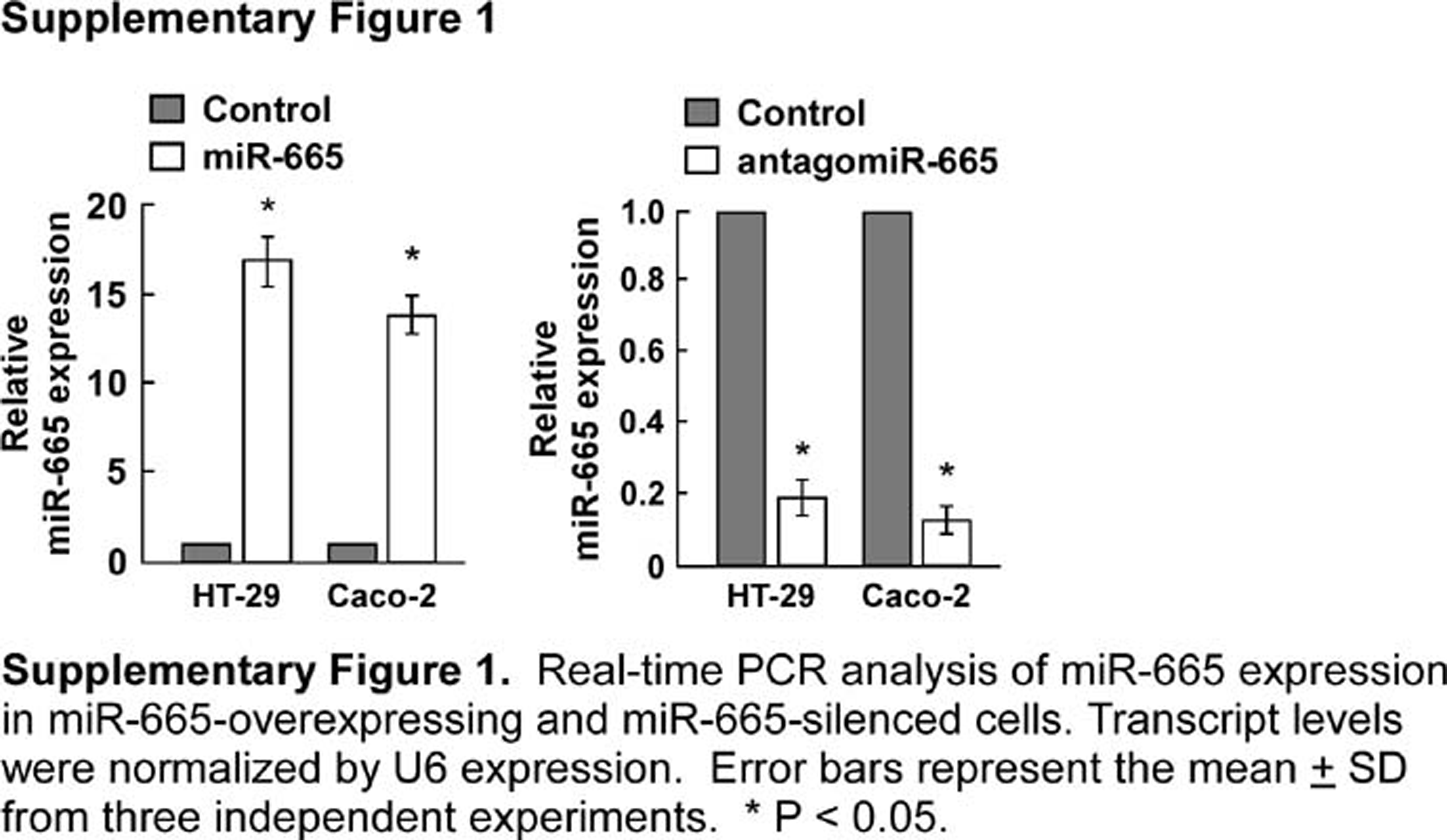

Supplement: Supplementary Figure 1 [file cddis201776x1.tif]

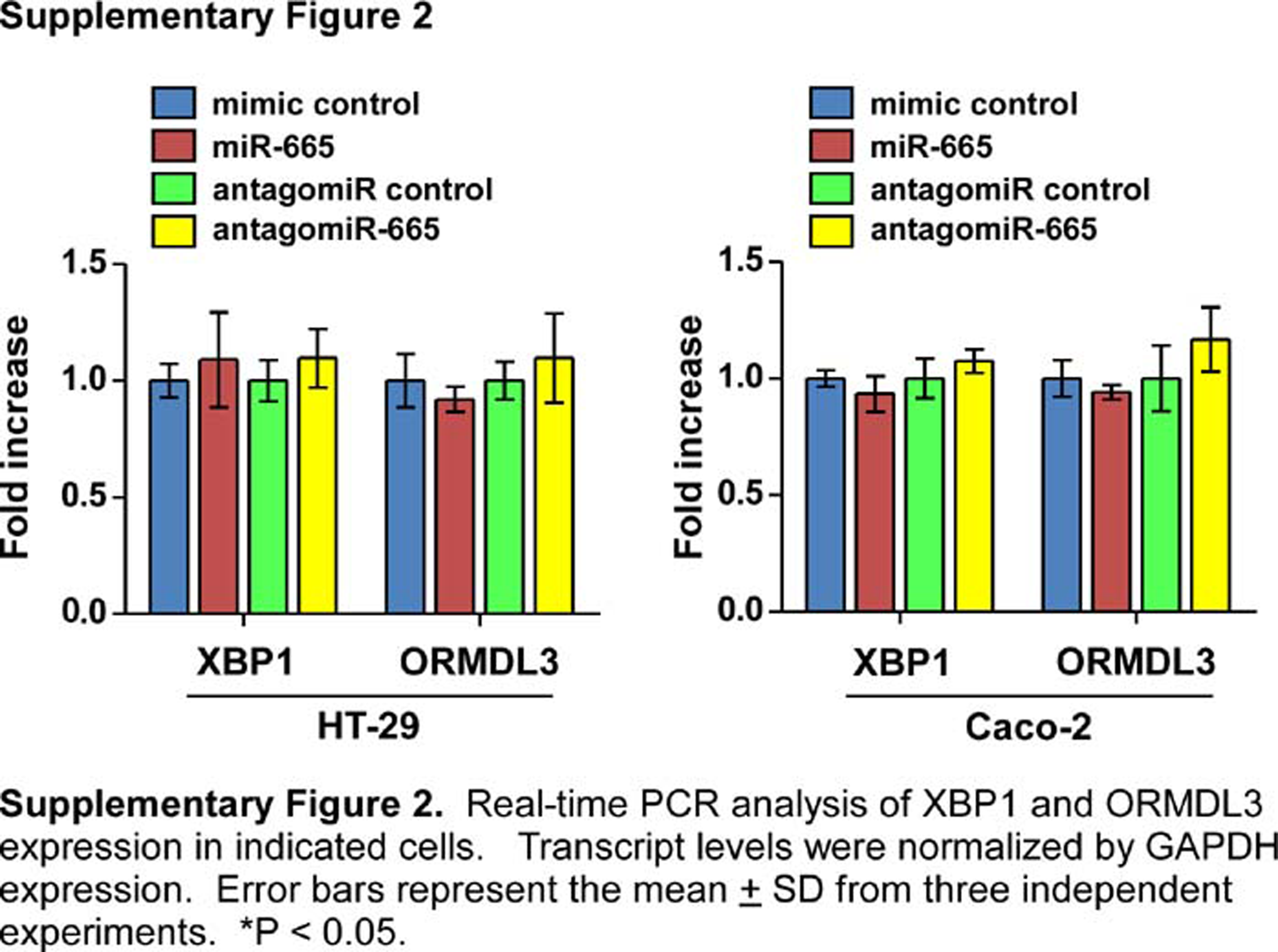

Supplement: Supplementary Figure 2 [file cddis201776x2.tif]

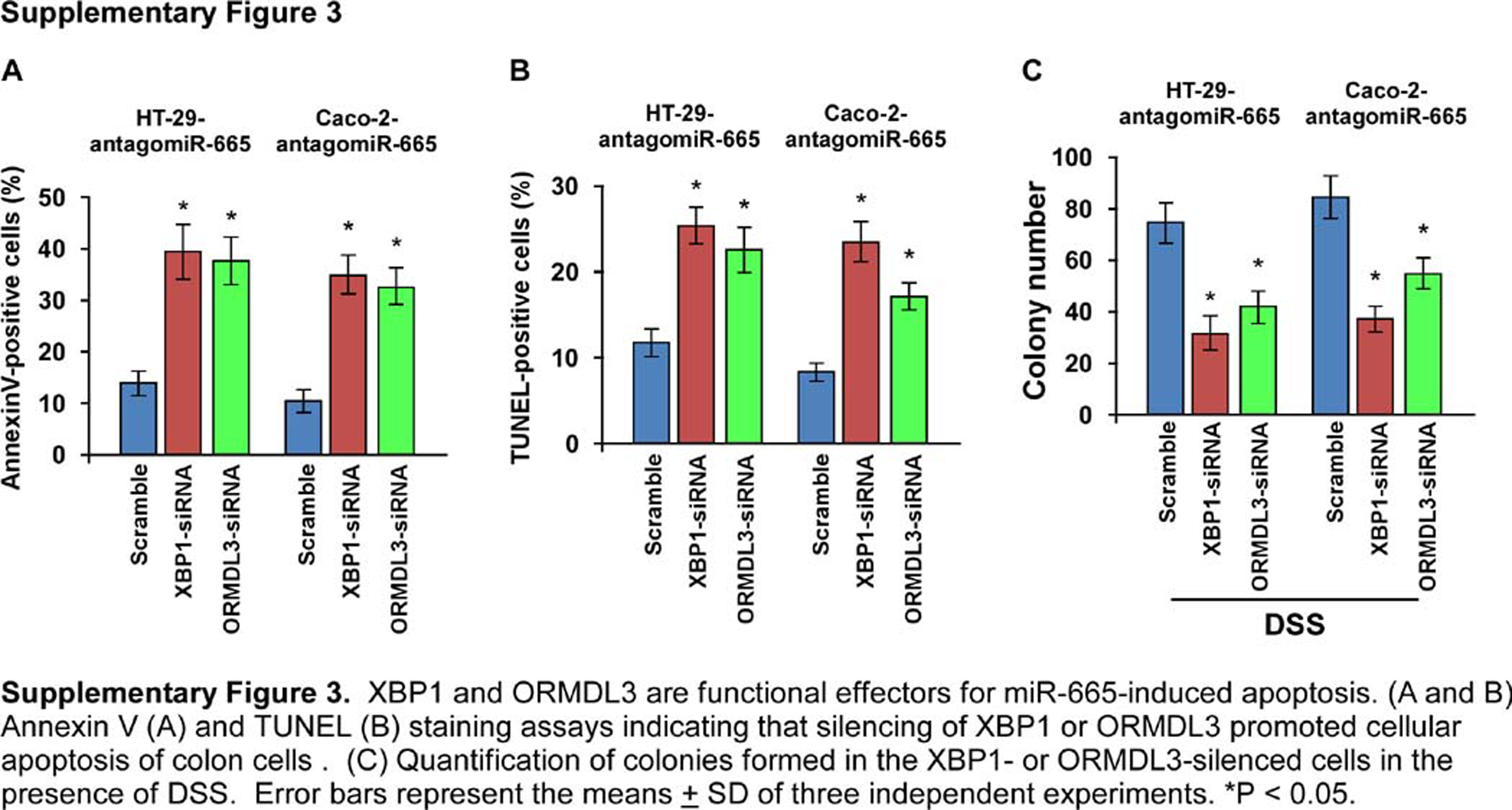

Supplement: Supplementary Figure 3 [file cddis201776x3.tif]
